# Funding for Chagas Disease: A 10-Year (2009–2018) Survey

**DOI:** 10.3390/tropicalmed5020088

**Published:** 2020-06-01

**Authors:** Leandro S. Sangenito, Marta H. Branquinha, André L. S. Santos

**Affiliations:** 1 Laboratório de Estudos Avançados de Microrganismos Emergentes e Resistentes (LEAMER), Departamento de Microbiologia Geral, Instituto de Microbiologia Paulo de Góes (IMPG), Universidade Federal do Rio de Janeiro (UFRJ), Rio de Janeiro 21941-901, Brazil; ibastefano@hotmail.com (L.S.S.); mbranquinha@micro.ufrj.br (M.H.B.); 2 Programa de Pós-Graduação em Bioquímica (PPGBq), Instituto de Química, Universidade Federal do Rio de Janeiro, Rio de Janeiro 21941-902, Brazil

**Keywords:** Chagas disease, human illness, neglected tropical disease, global financing, annual funding, investments

## Abstract

Chagas disease was discovered in 1909 by the Brazilian scientist Carlos Chagas. After more than 110 years, many outcomes have been achieved in all research fields; however, Chagas disease remains a serious public health problem, mainly in Latin America, being one of the most neglected tropical diseases in the world. As a neglected disease, it receives very little financial support. Nevertheless, how much is actually spent? With this question in mind, the goal of the present work was to summarize all funding employed by multiple institutions in the Chagas disease field in a 10-year survey. From 2009 to 2018, Chagas disease received only USD 236.31 million, representing 0.67% of the total applied for all neglected diseases in this period. Mostly, the investments are concentrated in basic research (47%) and drug development (42.5%), with the public sector responsible for 74% of all funding, followed by the industry (19%) and philanthropy (7%). Relevantly, NIH (USA) alone accounted for more than half of the total investment. Taking into account that Chagas disease has a great socio-economic impact, it is clear that more investments are needed, especially from endemic countries. Furthermore, coordinated strategies to make better use of resources and incentives for the pharmaceutical industry must be adopted.

## 1. Chagas Disease: An Overview

The American trypanosomiasis, or Chagas disease, is listed by the World Health Organization (WHO) as one of the 20 neglected tropical diseases (NTDs) [1,2]. The WHO estimates that 6 to 8 million people are infected with *Trypanosoma cruzi*, the etiologic agent of Chagas disease, and about 12 to 14 thousand deaths are reported every year (4.5 thousand only in Brazil). If left untreated, Chagas disease also can lead to severe digestive and heart system problems over the years [3]. Furthermore, more than 70 million individuals are living in areas with constant risk of transmission. The main form of contagion is through the blood-sucking triatomine insect bite; however, other forms of transmission are also reported, such as blood transfusions or organ/bone marrow transplants without proper control, congenital transmission (mainly in urban and non-endemic areas), laboratorial accidents, and orally, by ingesting food or liquids (e.g., açaí berry, palm wine, and sugar cane and guava juices) contaminated with triatomine feces containing infective *T. cruzi* metacyclic trypomastigotes [3,4]. This situation could have been worse if large-scale investments did not begin in the 90 s in order to promote interventions to interrupt the transmission. Important successful examples of multinational collaborations in Latin America must be exalted, such as the Southern Cone, Amazon, Andean and Central America initiatives. The common goals of these coordinated programs were to eradicate the main triatomine vectors of *T. cruzi* (*Rhodnius prolixus*, *Triatoma infestans*, *T. brasiliensis* and *T. dimidiata*) in combination with mandatory serological blood-bank screening [5,6,7,8]. Indeed, as vector and blood transfusion transmission has been combated in many countries, the incidence of Chagas disease has dropped substantially over the last decades [9].

**Figure 1:**
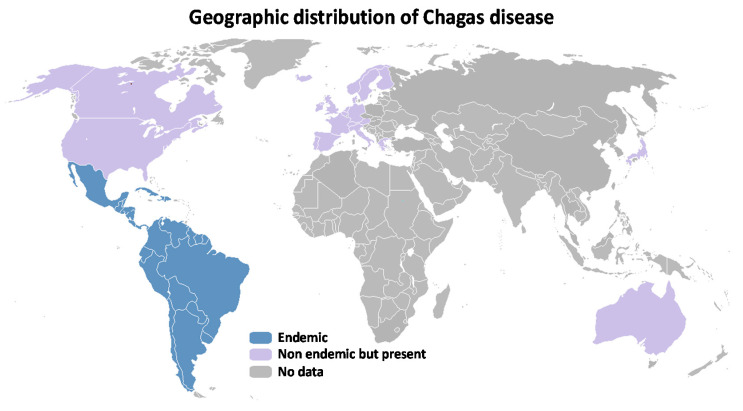
Global distribution showing the countries endemic for Chagas disease, where transmission occurs mainly through insect vector action, as well as the countries that receive infected people from these regions due to the migratory process [3,10,11].

Chagas disease is endemic in 21 countries of Latin America (about 6 million cases), but, due to the immigration, it is also reported in developed countries such as United States of America (USA) and some countries in Europe, such as Italy, Spain and France, representing a significant economic burden to the health care systems of these regions [3,10,11] (Figure 1). In Latin America, Argentina, Bolivia, Brazil and Mexico are in the top in terms of prevalence rates, accounting for approximately 70% (4.2 million) of the estimated cases of infected people [4,12].

There are several concerns about coping with this disease: besides the lack of an available vaccine, it is estimated that less than 10% of the people in the world affected by Chagas disease are diagnosed and only about 1% have access to specific treatment. The current treatment options, benznidazole and nifurtimox (the latter used in a smaller scale), have doubtful efficacy and require continuous monitoring. Moreover, these compounds present high manufacturing costs and they are extremely toxic, causing several side effects due to the necessity of long-term administration, which may require premature treatment interruption [13,14].

Despite the high morbidity and mortality rates and high costs of hospitalization and treatment, it has been clear that the pharmaceutical sector does not have any serious interest in financing specific research against Chagas disease, as it has for other chronic illness. Unfortunately, it is likely that this scenario has occurred because the population affected by this trypanosomiasis lives in poverty and, therefore, little financial return is expected with the development of diagnostic tests and new chemotherapeutic options. In part, this historical negligence contributes to the increase of disease morbimortality as well as to the spread poverty, and generates more social stigma [2,14,15,16].

The global impact of Chagas disease can be measured by Disability-Adjusted Life-Years (DALYs), an indicator that takes into account premature deaths and long-term irreversible injuries owing to the disease [17]. Per year, an impressive number, about 700,000, of DALYs has been attributed to Chagas disease, resulting in an overall cost of more than USD 7.2 billion [10,18]. The highest economic impact occurring outside Latin America is attributed to the USA, with an estimated average cost of USD 850 million. Inside Latin America, in Brazil alone, the annual economic losses are estimated at over USD 1.5 billion [10,18]. At the early stages of the disease, the estimated cost per patient is USD 200, but at the chronic symptomatic stage, this value can reach USD 4000 to USD 6000 [19].

For all the reasons mentioned, it is evident that Chagas disease has a huge social and economic global impact. Based on this scenario, it is clear that investments against the disease are extremely necessary. However, how much is actually applied and what is the profile of these investments? In this context, the present work aimed to briefly compile the investments applied to Chagas disease in a 10-year period (2009–2018), as well as the flow of them between the products and the main funders.

## 2. Data Collection for the Survey

This work was elaborated by compiling information collected from the repository of investment data provided by the G-FINDER project, conducted by Policy Cures Research, a not-for-profit global health think tank funded by the Bill & Melinda Gates Foundation [20]. The G-FINDER project groups annual funding data on neglected diseases provided by hundreds of institutions of governmental, private and philanthropic sectors. The survey was conducted from 2009 to 2018 (10 years) considering funding applied exclusively on Chagas disease, thus excluding investments in research and development (R&D) for multiple trypanosomiasis (Chagas disease, leishmaniasis and sleeping sickness). The year 2019 was not included in this work as data collection has not yet been completed. Pharmaceutical industry funding is presented aggregated for confidentiality reasons. For a better comparison of annual changes, funding data of all years were adjusted for the 2018 inflation rate and presented in US dollars (USD) to eliminate artefactual effects caused by inflation and exchange rate fluctuations.

## 3. Funds Applied to Chagas Disease over 10 Years

According to the data collected in the survey, the total amount of funds for R&D on neglected diseases reached almost USD 35 billion in a period of 10 years (2009–2018). The global investment in neglected diseases changed little over these years, with the exception of the year 2018, when the investment brand passed the USD 4 billion mark (Table 1). The majority of the global investments (USD 25 billion or 71.71%) were directed to only three diseases: AIDS, tuberculosis and malaria. In fact, this proportion is expected, since this “evil triad”corresponded to almost 276 million registered cases worldwide in 2018 (37.9 million of AIDS, 10 million of tuberculosis and 228 million of malaria) [21,22,23]. In 2018, the number of malaria deaths stood at 405 thousand (of these, an incredible 272 thousand were deaths of children under 5 years of age). The African region continues to carry a disproportionately high share of the global malaria burden, with 93% of malaria cases and 94% of malaria deaths [21]. Similarly, the African region accounts for more than two-thirds (25.7 million) of all people living with AIDS. The remaining (12.2 million) cases are spread across the world, but mainly in Eastern Europe and Central Asia. In 2018, 770 thousand people died from AIDS-related causes [22]. Tuberculosis is a treatable and curable disease. Even so, 1.5 million people died in 2018 (including 251 thousand people co-infected with HIV). A terrifying number of child deaths, 205 thousand, was also reported due to the tuberculosis (including among children infected with HIV). Tuberculosis is present in every part of the world. Therefore, the largest number of new cases in 2018 occurred in the South-East Asian region, with 44% of new cases, followed by the African region, with 24% of new cases and the Western Pacific with 18% [23]. Consequently, the high investment earmarked for this triad is easily justified by, collectively, the large number of cases worldwide, the global spread of the diseases and both morbidity and mortality rates, which mainly affect poor and developing countries as well as specific groups living in developed countries.

Unfortunately, investments in Chagas disease do not reflect the reality described above. In 10 years, the amount invested was USD 236.31 million, only 0.67% of the total applied for neglected diseases overall (Table 1). Both the number of infected people and the deaths associated with Chagas disease are inaccurate and probably underestimated [4]. For example, Chagas disease received USD 20.95 millionin 2018, which represents only from USD 2.6 to USD 3.5 per infected person and from USD 1496 thousand to USD1745 thousand per death. The investments combined for malaria, tuberculosis and AIDS in 2018 summed to USD 2.8 billion, representing about USD 10.2 per infected person (2.9 to 3.9 higher than for Chagas disease) and USD 1155 thousand per death (USD 340 to USD 590 less than Chagas disease). The “evil triad” has a higher lethality rate than Chagas disease; for this reason, the amount employed by death ends up being lower in view of the greater number of deaths. In this comparison, despite the lower lethality rate, the chronic form of Chagas disease is considered a disabling illness responsible for the most significant morbidity and mortality among parasitic diseases, in addition to great social stigma [4]. Indeed, as already mentioned, 700,000 DALYs have been attributed to Chagas disease each year (while DALYs for malaria in Latin America were estimated at 111,000), with a global cost for health systems exceeding the USD 7 billion mark, which reinforces the constant need for attention to this illness [2,4,12]. Therefore, appropriated investments in health care interventions should be framed in terms of the long-term savings to healthcare systemsas well as the economy [24].

In another comparison, other trypanosomatid diseases, such as leishmaniasis and sleeping sickness (or HAT, human African trypanosomiasis), also received much more funding in 10 years than Chagas disease: respectively, USD 503 million and USD 425 million. Leishmaniasis is endemic in approximately 98 countries worldwide, but especially located in Latin America, East Africa and Southeast Asia, with 14 million people directly affected by the three forms of the disease (cutaneous, mucocutaneous and visceral). By far, visceral leishmaniasis is the most important form, because it causes the most severe disease, with 200–400 thousand cases per year with 10%–20% mortality [25]. Moreover, leishmaniasis is also of great importance in the context of veterinary medicine: dogs have great domestic appeal as well as being the main reservoirs for several *Leishmania* species. Therefore, it should be noted that this dichotomy is responsible for part of the USD 38.87 million investment destined to the disease in 2018 [26]. HAT, on the other hand, is a more restricted disease, affecting mainly African countries such as the Democratic Republic of the Cong, which is responsible for 70% of the cases reported in the last 10 years. Although fewer than 977 cases were reported in 2018 in endemic countries, HAT is still a public health problem in endemic regions, receiving USD 50.63 million of investments in the same year [27,28]. In general, investments destined for Chagas disease summed to around the USD 20 million mark. Three years stood out: 2011, with USD 27.03 million, 2012, with USD 35.28 million, and 2013 with USD 28.3 million. Notably, in 2012, the percentage directed to American trypanosomiasis managed to slightly pass the mark of 1% of the total applied for neglected diseases in the same year (Table 1). In 2008, Chagas disease received an investment of around USD 18 million. It is interesting to note that, even with the huge world economic crisis of 2008 [29], investments in Chagas disease continued to rise, reaching theirpeak in 2012, when they started to stabilize (Table 1).

R&D funding for Chagas disease is largely concentrated in basic research (47%) and development/repositioning of drugs (42.5%). On a second level, the development of diagnostics and vaccines accounts for only 10% of funding flow. All remaining products (biologics, vector control and others) received less than 1%. In 2009 and 2010, basic research received about three times the total applied for the development of new therapies. Thereafter, the flow of financing for drug products received an extraordinary increase in 2011 (up to USD 6.31 million, which corresponds to 114.1%), even surpassing the basic research funding in the subsequent years 2012, 2013, 2014 and 2015. In 2017, the funds for drugs went back to the level of earlier years due to many cuts (Table 2). In part, the Brazilian Support Foundation for Research in the State of São Paulo (FAPESP) dropped out of the top main funders because no funding was reported for Chagas disease R&D in 2017, due to the steep cuts in Brazilian public agencies’ spending. In 2018, a notable increase in funding for drug R&D (up USD 6.87 million, which corresponds to 154.7%), occurred again, as a result of record high industry investment and the Mundo Sano Foundation’s USD 1.8 million grant to the Drugs for Neglected Diseases *initiative* (DND*i*) for pediatric benznidazole to treat Chagas disease.

**Figure 2:**
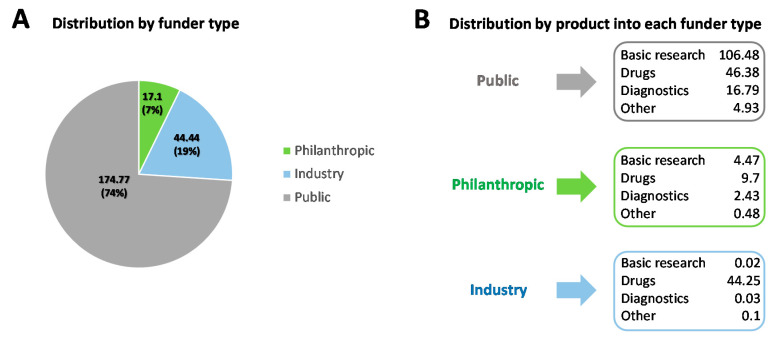
(**A**) Funds invested in Chagas disease for 10 years by the three main investment sectors. (**B**) Funds from the three main investment sectors distributed by product each type [20]. The values are adjusted for 2018 inflation and are expressed in US million dollars.

The responsibility for investing in Chagas disease falls mainly on the shoulders of the public sector. In 10 years, governmental institutions were responsible for 74% of total funding (USD 174.77 million). In the second place, industry funding reached 19% of total funding (USD 44.44 million), followed by philanthropic institutions with 7% (USD 17.1 million) (Figure 2A). If only the funding from public sectoris taken into account, 60.9% (USD 106.48 million) was applied to basic research. This amount represents 96% of total funding designated for basic research in 10 years, demonstrating the great dependence of this type of research on the public sector. Here, we emphasize that the investment in basic research includes all investment for Chagas disease/*T. cruzi*. However, although much research deals with the pathogen that causes the disease, this research may not have a direct influence on combating the disease itself. The public sector also significantly funds drug development/repositioning, designating 26.5% (USD 46.38 million) of its total investments. Although the public sector allocates “only” about a quarter of its entire investment in drug products, this total, USD 46.38 million, represents about 46.2% of the total investment in drugs over 10 years. Another 44.1% (USD 44.25 million) of the total funds for drug products came from the aggregate industry. It is also interesting to note that this portion basically represents the whole private sector investment. The remaining 9.67% of total drug funds (USD 9.7 million) were donated by philanthropic organizations, which also represented the main destination of resources (56.7%) from this type of funder. Finally, public agencies were also responsible for funding 87.3% of total investments in diagnosis. The remaining funds (12.6%, USD 2.43 million) came almost entirely from philanthropy (Figure 2B).

**Figure 3:**
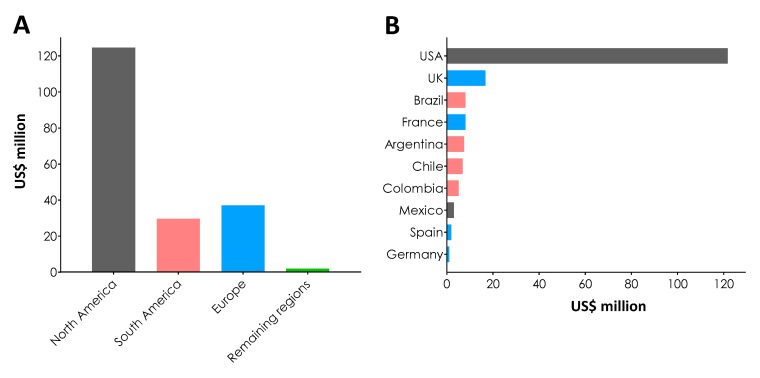
(**A**) Funds for Chagas disease from the main geographic regions. (**B**) Fractioned funds by each country. The colors are correlated with each geographic region in (**B**). The data in (**A**) and (**B**) refer to investments coming from the public sector and philanthropic organizations in each region, thus excluding the private sector, which has headquarters in different countries. The values are adjusted for 2018 inflation and are expressed in USD in million. Note that the countries are grouped by their geographic regions and not by the origin of the spoken language (like the Latin American countries); in this way, Mexico is found in North America [20].

In a list of more than 50 R&D funders of all types, the top 20 accounted for 96.8% of all R&D funding for Chagas disease over 10 years. However, the top three funders alone—the US NIH, industry, and the Wellcome Trust—provided nearly three-quarters (USD 180.26 million, 76.3%) of all funding (Table 3). If the funds over 10 years are separated by geographic regions, excluding the aggregate industrial sector, investors in North America are responsible for the majority (USD 124.3 million) of R&D financing of Chagas disease. Within these, the public sector of the American government, represented by the NIH, was solely responsible for investing USD 119.79 million of that amount, with the remainder practically coming from Mexico (Figure 3A, Table 3). Interestingly, Europe is responsible for the second-largest portion of all investments in Chagas disease, about USD 36.7 million. Within this, USD 17.84 million (48.6%) was destined to basic research, followed by USD 15.32 million (41.74%) for drug development/repositioning and USD 2.91 million (7.93%) for diagnostic products. This huge investment is due to the great contribution of the philanthropic institution the Wellcome Trust, the European commission, and Institute Pasteur, which reflects the European concern with Latin American immigrants who arrive infected with *T. cruzi* and become a public health problem for their governments (Figure 3A, Table 3) [3,30]. Indeed, Basile and coworkers [31] estimated the number of *T. cruzi*-infected immigrants living in Europeas ranging between 68,318 and 123,078, with 4290 confirmed cases, 94%–96% remaining undiagnosed. This scenario stimulated several meetings to address the problem, leading to recommendations, formation of coordinated working groups and adoption of various control measures [3,30]. Finally, South America, the region with the vast majority of cases, is the geographic region providing thethird-most R&D funds (Figure 3). This fact should be seriously reviewed, since Chagas disease is mainly a South American problem. Therefore, it is unacceptable that South America spends less to combat the illness than Europe, despite the fact that the gross domestic product of some South American countries is comparable to some European countries [32].

The top 10 countries that invest in Chagas disease are listed in Figure 3B. By far, the USA appears as the major funder, contributing USD 121.45 million. In second place is the United Kingdom (UK), contributing USD 16.45 million, where 97.4% (USD 16.03 million) came from the generosity of the philanthropic institution the Wellcome Trust. Brazil appears in the third position, followed very closely by France, Argentina and Chile (Figure 3B, Table 3). It is terrifying to note that Bolivia, one of the main countries in terms of cases and risk of infection [4,12], does not figure in the list of the main funders of R&D to control American trypanosomiasis, perhaps due to the lack of participation of the country’s agencies in providing data.

The major change in the last fifteen years in the Chagas disease landscape has been the appearance of new investigators and initiatives from the pharmaceutical industry, academic groups, and consortia, encouraging and promoting further R&D in the area [33]. In future years, global financial contributions against Chagas disease will also come from a new partner. At the end of 2019, the international center for the Purchase of Medicines against AIDS, malaria and tuberculosis (UNITAID), an entity created by Brazil, Chile, France, Norway and UK, announced that it will contribute, for the first time, USD 20 million over four years [34]. The idea is to develop strategies and tools to improve prevention and diagnosis, reduce congenital infection, and provide faster treatments and drug formulations with fewer side effects than the nitroderivatives. The expectation of the entity, which is also a partner of WHO, is that research institutions of excellence in more than one country will establish new consortia to seek solutions. Indeed, collaborative strategies between several health and research institutions, such as the DND*i*, are a smart bet, which save time and costs and can produce relevant results [16]. Therefore, they must always be considered.

## 4. Topics to Be Addressed on Chagas Disease Funding

For an illness surrounded by stigma, affecting millions, funding for Chagas disease R&D is far from ideal. Although the last 15 years have seen considerable evolution, there are still several issues to be worked on to overcome the problems associated with limited treatment [4,33]. New advances in R&D from recent clinical trials have been made in the last decade; however, it is urgent to review the current evidence and define future research priorities. A focused and collaborative effort from the entire research community would ensure the development of appropriate and efficacious anti-*T. cruzi* drugs [24]. Collaborative efforts are also welcome for developing other products and pursuing important strategies. For instance, vaccine candidates for Chagas disease are at the pre-clinical stage [35], representing a future front in the battle. Moreover, it is also crucial to scale efforts up to provide easier access to diagnostic testing and medical therapy for chagasic patients. Finally, the designation of resources to triatomine vector control was a rational and the cost-effective approach to drastically reduce the incidence of the disease, but continuation of these public health programs is needed to maintain success [2,36]. While increasing spending may cause acute strain on research budgets, it is clear that improving preventative efforts on Chagas disease can effectively reduce this spending over time [24].

Usually, development agencies focus their spending majority on operational and field research, not R&D. This statement also appears at the basic level of global health planning with the premise that new technologies would divert attention and funding away from real concerns [36]. Global health agencies, including the WHO, do not consider R&D a core indicator that the global community prioritizes. They take the position that scarce funds should be primarily spent on traditional public health issues such as infrastructure, the workforce, health data and health services. This is extremely unfounded and unhelpful since promising innovation does not compete with program health funding. The initial cost of R&D, which seems high during the innovation process, becomes smaller and smaller over decades of use, especially when set against the saving of lives and health resources [36]. The imperative for public health programs was to prioritize the resources already available, employing low-cost tools for many neglected health problems (more than 95% of drugs on the WHO’s Essential Medicines List were older off-patent drugs) [37]. Moreover, pharmaceutical companies seek firstly profit maximization, not global public health improvement. To contextualize, of the 1556 new compounds brought to market between the period from 1975 to 2004, only 20 (1.28%) were for neglected diseases, and none of them for Chagas disease [38]. Even with little investment in innovation, it is no surprise that all of these 20 compounds were developed with public-sector involvement [2]. It is critical that the international community take it upon themselves to address the predictable gap in R&D (that disproportionately affects developing countries) conducted by the pharmaceutical industry. However, the public sectors also cannot exempt themselves from responsibility and must promote actions that maximize results as well as encourage the private sector to supportthe cause.

To address the structural R&D gap, coordinated strategies should be employed between global health and development agencies in order to produce new tools and treatments for Chagas disease. For this, the maintenance of a medical R&D agenda driven by Chagas disease needs is crucial, besides serious international and local financing to support this R&D, not just for traditional operational and health systems research. Governments should also cautiously promote market-based incentives that seek to stimulate pharmaceutical R&D. It is also important torapidly increase innovation expertise (with open access to the resultant knowledge) in order to achieve effective R&D prioritization and smart funding decisions. Moreover, all subjects of the equation need to engage far more closely with each other, to secure better sequencing of their activities and a better strategic alignment between what the Chagas disease community needs and what the science community does [2,24,36].

## 5. Conclusions

The present work summarized the investments applied to Chagas disease over a period of 10 years. As we have seen, little funding has been directed towards to this specific parasitic illness when compared to other neglected diseases. For a human disease that is responsible annually for about 700,000 DALYs, with an economic impact of more than USD 7 billion, receiving just 0.67% (USD 236.3 million) of the total budget for neglected diseases (considering the period from 2009 to 2018) is unacceptable and meaningless. Therefore, allocating more funds to fight Chagas disease is urgently necessary in terms of saving lives, restoring quality of life and reducing the economic impact coming from health care systems.

Regarding Chagas disease, clearly, funding depends much more on the public sector (74%) than on other areas (private and philanthropic). However, public agencies in Latin American countries contribute very little compared to NIH-USA, a situation that needs to be reviewed. In addition, development agencies, governments and the Chagas disease community must work in unison to ensure that the (scarce) resources are well applied. In parallel, there is a need to adopt strategies and market incentives to attract the pharmaceutical industry more to the Chagas disease cause, since the sector contributed only USD 44.4 million over 10 years. Other relevant issues to be addressed include the low investment in important tools such as diagnostics, vaccines and vector control, which together received just 10% of the total budget, with the remaining equally distributed in basic research and drug development/repositioning.

Finally, we hope that this survey helps to support the work of many other groups in the Chagas disease community, facilitating decision-making and even more effective application ofthe available resources.

## Figures and Tables

**Table 1:** Investments in neglected diseases over 10 years [20].

Investments in Neglected Diseases in 10 Years (USD in Million *)												
Diseases	2009	2010	2011	2012	2013	2014	2015	2016	2017	2018	Total	%
**Neglected Diseases**	3595	3416	3364	3469	3348	3337	3282	3437	3681	4055	34,984	100
**Chagas Diseases**	19.31	23.3	27.03	35.28	28.3	21.65	19.15	23.4	17.94	20.95	236.31	0.67

* Adjusted values for 2018 inflation.

**Table 2:** Chagas disease R&D funding [20].

Chagas Disease R&D Funding in 10 Years (USD in Million *)											
Areas	2009	2010	2011	2012	2013	2014	2015	2016	2017	2018	Total by Product
**Basic Research**	13.47	14.07	11.74	14.26	9.82	8.14	8.12	12.06	11.48	7.78	110.94
**Drugs**	4.38	5.53	11.84	17.54	16.41	11.64	9.21	8.02	4.44	11.31	100.32
**Diagnostics**	0.9	2.79	3.15	3.01	1.63	1.24	1.09	2.0	1.67	1.75	19.23
**Vaccines**	0.53	0.86	0.26	0.26	0.21	0.42	0.63	0.92	0.26	0.02	4.37
**Biologics**	NA	0.02	0.01	NA	NA	0.02	<0.01	0.26	0.02	0.02	0.35
**Vector Control**	<0.01	NA	NA	0.01	0.03	NA	NA	0.06	0.05	0.04	0.2
**Other**	0.02	0.01	0.01	0.18	0.2	0.17	0.09	0.04	0.02	0.03	0.77
**Total by Disease**	19.31	23.3	27.03	35.28	28.3	21.65	19.15	23.4	17.94	20.95	

* Adjusted values for 2018 inflation; NA, not applied.

**Table 3:** Top 20 funders in Chagas disease [20].

Top 20 Funders in Chagas Disease (USD in Million *)			
1. US NIH	119.79	11. Brazilian FAPESP	2.45
2. Aggregate Industry	44.44	12. French IRD	2.16
3. Wellcome Trust	16.03	13. Mundo Sano Foundation	1.85
4. European Commission	8.9	14. Carlos III Health Institute	1.27
5. Chilean FONDECYT	6.04	15. LAFEPE	1.25
6. Colombian Colciencias	4.85	16. Brazilian FAPEMIG	1.14
7. Institute Pasteur	4.54	17. Brazilian BNDES	1.11
8. Argentinian MINCYT	4.4	18. French ANR	1.04
9. Brazilian DECIT	3.15	19. Argentinian CONICET	0.93
10. Mexican CONACYT	2.48	20. Gates Foundation	0.83

* Adjusted values for 2018 inflation.

## References

[1] (2020). World Health Organization. https://www.who.int/neglected_diseases/diseases/en/.

[2] Crager S.E., Price M. (2009). Prizes and Parasites: Incentive Models for Addressing Chagas Disease. J. Law Med. Ethics.

[3] Lidani K.C.F., Andrade F.A., Bavia L., Damasceno F.S., Beltrame M.H., Messias-Reason I.J., Sandri T.L. (2019). Chagas disease: From discovery to a worldwide health problem. Front. Public Health.

[4] Pinheiro E., Brum-Soares L., Reis R., Cubides J.C. (2017). Chagas disease: Review of needs, neglect, and obstacles to treatment access in Latin America. Rev. Soc. Bras. Med. Trop..

[5] Schofield C.J., Dias J.C. (1999). The Southern Cone Initiative against Chagas disease. Adv. Parasitol..

[6] Guzmán-Bracho C. (2001). Epidemiology of Chagas disease in Mexico: An update. Trends Parasitol..

[7] Guhl F., Restrepo M., Angulo V.M., Antunes C.M., Campbell-Lendrum D., Davies C.R. (2005). Lessons from a national survey of Chagas disease transmission risk in Colombia. Trends Parasitol..

[8] Hashimoto K., Schofield C.J. (2012). Elimination of *Rhodnius prolixus* in Central America. Parasites Vectors.

[9] Chuit R., Meiss R., Salvatella R., Altcheh J., Freilij H. (2019). Epidemiology of Chagas Disease. Chagas Disease.

[10] Lee B.Y., Bacon K.M., Bottazzi M.E., Hotez P.J. (2013). Global economic burden of Chagas disease: A computational simulation model. Lancet Infect. Dis..

[11] World Health Organization (2015). Chagas disease in Latin America: An epidemiological update based on 2010 estimates. Wkl. Epidemiol. Rec. (WER).

[12] World Health Organization (2015). Investing to overcome the global impact of neglected tropical diseases: Third WHO report on neglected diseases. Libr. Cat. Publ. Data.

[13] Sales P.A., Molina I., Fonseca M.S.M., Sánchez-Montalvá A., Salvador F., Corrêa-Oliveira R., Carneiro C.M. (2017). Experimental and clinical treatment of Chagas disease: A review. Am. J. Trop. Med. Hyg..

[14] (2019). Drugs for Neglected Diseases Initiative. https://www.dndi.org/wp-content/uploads/2019/09/Factsheet2019_ChagasDisease.pdf.

[15] Bhutta Z.A., Sommerfeld J., Lassi Z.S., Salam R.A., Das J.K. (2014). Global burden, distribution, and interventions for infectious diseases of poverty. Infect. Dis. Poverty.

[16] Sangenito L.S., da Silva S.V., d’Avila-Levy C.M., Branquinha M.H., Santos A.L.S., Oliveira S.S.C. (2019). Leishmaniasis and Chagas disease—neglected tropical diseases: Treatment updates. Curr. Top. Med. Chem..

[17] Dias L.C., Dessoy M.A., Silva J.J.N., Thiemann O.H., Oliva G., Andricopulo A.D. (2009). Chemotherapy of Chagas’ disease: State of the art and perspectives for the development of new drugs. Quim. Nova.

[18] Ferreira L.G., de Oliveira M.T., Andricopulo A.D. (2016). Advances and progress in Chagas disease drug discovery. Curr. Top. Med. Chem..

[19] Abuhab A., Trindade E., Aulicino G.B., Fujii S., Bocchi E.A., Bacal F. (2013). Chagas’ cardiomyopathy: The economic burden of an expensive and neglected disease. Int. J. Cardiol..

[20] G-Finder Project. https://gfinderdata.policycuresresearch.org/.

[21] (2020). Malaria Fact Sheets. https://www.who.int/news-room/feature-stories/detail/world-malaria-report-2019.

[22] (2020). AIDS Fact Sheets. https://www.who.int/news-room/fact-sheets/detail/hiv-aids.

[23] (2020). Tuberculosis Fact Sheets. https://www.who.int/en/news-room/fact-sheets/detail/tuberculosis.

[24] Echeverría L.E., Marcus R., Novick G., Sosa-Estani S., Ralston K., Zaidel E., Forsyth C., Ribeiro L.P., Mendoza I., Falconi M.L. (2020). WHF IASC Roadmap on Chagas Disease. Glob. Heart.

[25] (2020). Leishmaniasis Fact Sheets. https://www.who.int/news-room/fact-sheets/detail/leishmaniasis.

[26] Dantas-Torres F., Miró G., Baneth G., Bourdeau P., Breitschwerdt E., Capelli G., Cardoso L., Day M.J., Dobler G., Ferrer L. (2019). Canine Leishmaniasis Control in the Context of One Health. Emerg. Infect. Dis..

[27] (2020). Sleeping Sickness Fact Sheets. https://www.who.int/news-room/fact-sheets/detail/trypanosomiasis-human-african-.

[28] Gao J.M., Qian Z.Y., Hide G., Lai D.H., Lun Z.R., Wu Z.D. (2020). Human African trypanosomiasis: The current situation in endemic regions and the risks for non-endemic regions from imported cases. Parasitology.

[29] World Health Organization. https://www.who.int/topics/financial_crisis/financialcrisis_report_200902.pdf.

[30] Liu Q., Zhou X.N. (2015). Preventing the transmission of American trypanosomiasis and its spread into non-endemic countries. Infect. Dis. Poverty.

[31] Basile I., Jansà J.M., Carlier Y., Salamanca D.D., Angheben A., Bartoloni A., Seixas J., Van Gool T., Canavate C., Flores-Chavez M. (2011). Chagas disease in European countries: The challenge of a surveillance system. Euro Surveill..

[32] (2020). World Population Review: GDP Ranked by Country. https://worldpopulationreview.com/countries/countries-by-gdp/.

[33] Chatelain E. (2015). Chagas Disease Drug Discovery: Toward a New Era. J. Biomol. Screen..

[34] Brazil, Ministry of Health. https://www.saude.gov.br/noticias/agencia-saude/46058-brasil-emplaca-maior-investimento-mundial-contra-doenca-de-chagas.

[35] Beaumier C., Gillespie P., Strych U., Hayward T., Hotez P.J., Bottazzi M.E. (2016). Status of vaccine research and development of vaccines for CD. Vaccine.

[36] Moran M. (2016). The Grand Convergence: Closing the Divide between Public Health Funding and Global Health Needs. PLoS Biol..

[37] Saez C. WHO Reviews Its Essential Medicines List; Some New Candidates Under Patent. https://www.ip-watch.org/2015/04/21/who-reviews-its-essential-medicines-list-some-new-candidates-under-patent/.

[38] Moran M., Guzman J., Ropars A.L., McDonald A., Jameson N., Omune B., Ryan S., Wu L. (2009). Neglected disease research and development: How much are we really spending?. PLoS Med..

